# Vitamin D status in early childhood is not associated with cognitive development and linear growth at 6–9 years of age in North Indian children: a cohort study

**DOI:** 10.1186/s12937-020-00530-2

**Published:** 2020-02-10

**Authors:** Ranadip Chowdhury, Sunita Taneja, Ingrid Kvestad, Mari Hysing, Nita Bhandari, Tor A. Strand

**Affiliations:** 1 Centre for Health Research and Development, Society for Applied Studies, 45, Kalu Sarai, New Delhi, 110016 India; 2 Department of Global Public Health and Primary Care, Centre for International Health, University of Bergen, Bergen, Norway; 3 Regional Centre for Child and Youth Mental Health and Child Welfare, West, NORCE Norwegian Research Center, Bergen, Norway; 4 Department of Psychosocial Science, Faculty of Psychology, University of Bergen, Bergen, Norway; 5 Department of Research, Innlandet Hospital Trust, Lillehammer, Norway

**Keywords:** Vitamin D, Wechsler intelligence scale for children, 4th edition ^INDIA^, Crichton verbal scale, A developmental neuropsychological assessment II, The behavior rating inventory of executive function 2, Linear growth, School age

## Abstract

**Background:**

Vitamin D is important for brain function and linear growth. Vitamin D deficiency during pregnancy has been linked with impaired neurodevelopment during early childhood. However, there is limited evidence from population-based studies on the long-term impact of vitamin D deficiency on cognitive development and linear growth. The objective of the current analysis is to examine whether vitamin D deficiency during infancy and early childhood is associated with cognitive development and linear growth measured in school age.

**Methods:**

This is a follow-up study of a placebo-controlled trial among 1000 North Indian children 6–30 months of age. We measured growth and neurodevelopment in 791 of these children when they were 6–9 years old. Neurodevelopment was measured using the Wechsler Intelligence Scale for Children, 4th edition ^INDIA^, the Crichton Verbal Scale, NEPSY-II subtests, and the BRIEF 2. We categorized vitamin D concentrations during infancy and early childhood according to the US Institute of Medicine’s recommendations; serum 25(OH)D < 12 ng/ml as deficient; 12–20 ng/ml as inadequate; > 20 ng/ml as sufficient. In multivariable regression models, adjusting for relevant confounders, we estimated the association between vitamin D status, growth and neurodevelopmental outcomes.

**Results:**

Among the 791 children, baseline vitamin D status was available for 716. Of these, 45.8% were vitamin D deficient, 32.7% were inadequate, and 21.5% were sufficient. Vitamin D status was not associated with any of the cognitive outcomes or linear growth [Adjusted β coefficient for height for age z-score between deficient and sufficient children was − 0.06 (95% CI − 0.24 to 0.11)] at follow up.

**Conclusion:**

Our findings do not support the notion that poor vitamin D status in early childhood is an important limitation for cognitive development and linear growth.

**Trial Registration:**

The trial was first registered at www.clinicaltrials.gov as  NCT00717730 in July, 2008, and at CTRI/2010/091/001090 in August, 2010 and then as CTRI/2016/11/007494 in November 2016.

## Background

Vitamin D deficiency is one of the most common micronutrient deficiencies worldwide [1]. In the Indian subcontinent, the prevalence of vitamin D deficiency is estimated to be from 50 to 90% using an internationally accepted reference value [2]. Vitamin D acts by binding to the nuclear vitamin D receptors (VDR), which are widely distributed throughout the human brain in most neurons and some glial cells [3, 4]. Animal studies have shown that vitamin D deficiency during pregnancy causes extreme alterations in the brain at birth [5, 6]. This provides a biological plausibility for a link between vitamin D status and neurodevelopment.

There is evidence from observational studies on the association between vitamin D status during pregnancy or cord blood vitamin D at birth with cognitive, language, and behavioral development in different periods of childhood [7–12]. Previously, we have shown that vitamin D status was not associated with neurodevelopment as measured by a brief screening tool, the Ages and Stages Questionnaire 3rd edition (ASQ-3) during early childhood [13]. The consequences of vitamin D deficiency in early life on neurodevelopmental may not become evident until later in childhood. Furthermore, the predictive ability of early neurodevelopmental assessments is poor, and cognitive assessments in school-aged children have shown to be stable over time [14, 15]. We measured vitamin D status in 1000 young North Indian children and conducted a comprehensive assessment of cognitive performance and growth approximately 6 years later [16, 17]. This study gave us a unique opportunity to explore the extent to which vitamin D deficiency during early childhood is associated with impaired cognitive development and linear growth at school age.

## Methods

### Study design and participants

**Fig. 1:**
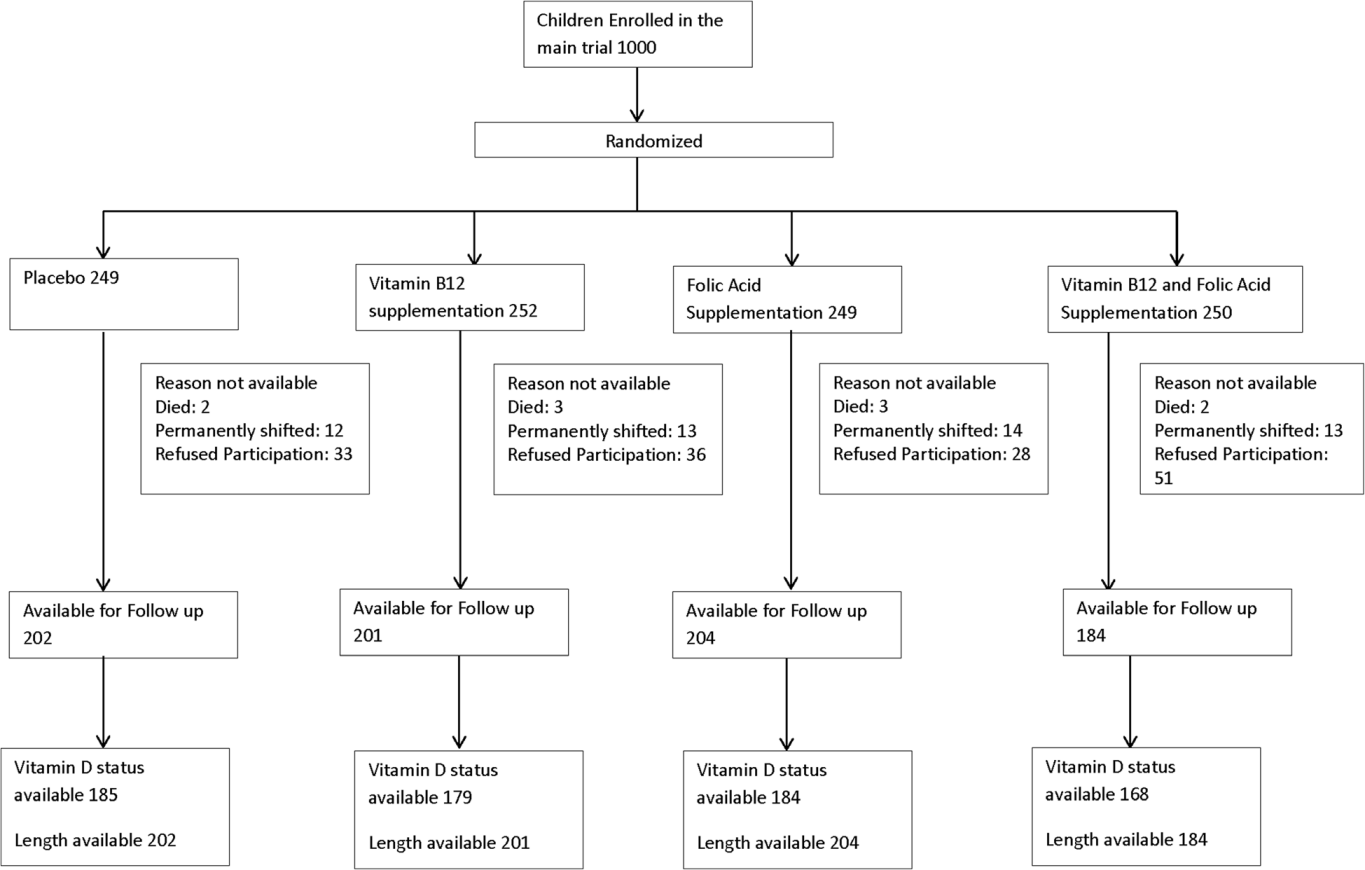
Participant Flow

We followed up children who previously participated in a randomized double-blind placebo-controlled trial (*n* = 1000) on the effect of two recommended daily allowances (RDA) of vitamin B12 and/or folic acid daily for 6 months in Delhi, North India [18]. The main outcome of the study was incidence of infections. In September 2016, we approached all these children, and were able to get in contact with 798 of whom 791 consented to participate in the follow-up study. (Fig. 1) All families were initially contacted by phone to be invited to participate in the study. A physical visit was made to the family’s address if no contact could be made. We requested the families who had moved out of the study area to come to the study clinic for 1 day. On the day of the assessment, consent was taken from the children’s caregiver for the participation in the study and we gathered information on socio-economic situation of the family such as parental education and occupation and various household assets.

### Assessment

#### Cognition

We assessed cognitive development with the Wechsler Intelligence Scale for Children, 4th edition ^INDIA^, the Crichton Verbal Scale, NEPSY-II subtests and the BRIEF 2.

The Wechsler Intelligence Scale for Children 4th edition (India) (WISC-IV^INDIA^) is an assessment tool of intellectual ability in children validated for the Indian population with Indian norms [19]. We assessed seven subtests (listed in the parenthesis) that were summed up to three index scores; the Perceptual Reasoning (Block design, Picture concept, Matrix reasoning), Processing Speed (Symbol search, Letter-number sequences) and Working Memory (Digit span, Coding).

The Crichton Vocabulary Scales (CVS) assess verbal skills in children 4 to 18 years through knowledge of words [20]. The CVS has been translated to Hindi and provides a standard score with Indian norms [21].

NEPSY-II is a neuropsychological test battery for children aged 3 to 16 years with American norms [22]. Seven age-appropriate subtests were administered; Inhibition and Design Fluency, Word Generation, Visuo-motor Precision and Manual Motor Sequences, Affect Recognition and Geometric Puzzles. No modifications and cultural adjustments were necessary to perform the tests in this setting.

The Behavior Rating Inventory of Executive Function 2nd edition (BRIEF 2) is a parent report questionnaire for children 5 to 18 years assessing executive functions in everyday life with American norms [23]. The scale comprises three clinical indexes; the Behavior, Emotion and Regulation Index, and an overall Global Executive Composite. The questionnaire was translated and validated to Hindi in close collaboration with the developers (PARiConnect).

### Growth

Trained field supervisors measured weight and height at follow up. Weight was measured to the nearest 50 g using a Digitron scales. Height was measured with Seca 213 to the nearest 0.1 cm. Inter- and intra-observer standardization exercises for weight and height assessments were conducted before study initiation for outcome ascertainment team; these are repeated every 3 months.

### Analytical procedures

At baseline, 3 ml blood was collected into an evacuated tube containing EDTA (BD, Franklin Lakes, NJ, USA) from all children. Plasma was separated from the whole blood by centrifugation at room temperature (450 x g × 10 min), transferred into storage vials and stored at − 20 °C until analysis. Plasma concentration of vitamin-D was measured by quantitative electro-chemiluminescence binding assay (Roche Diagnostics, Mannheim, Germany) at the Department of Biochemistry, Christian Medical College, Vellore, India [24]. Plasma homocysteine (tHcy) was analyzed using commercial kits (Abbott Park, IL, USA) [25]. Plasma concentrations of vitamin B12 and folate were determined by microbiological assays using a chloramphenicol-resistant strain of *Lactobacillus casei* and colistin sulfate-resistant strain of *Lactobacillus leichmannii*, respectivel y[26, 27]. Plasma soluble transferrin receptor (sTfR) was analyzed using an immunoturbidimetric assay [28].

### Statistical analysis

Proportions, means (SD) or medians (IQR) were calculated for categorical and continuous variables by vitamin-D status at baseline. We categorized vitamin D status according to the US Institute of Medicine’s recommendations; serum 25(OH)D < 12 ng/ml as ‘deficient’; 12–20 ng/ml as ‘inadequate’; > 20 ng/ml as ‘sufficient’ [29]. For the cognitive outcomes, we calculated a combined WISC-IV^INDIA^ and CVS z-score based on converted z-scores for the three index scores in the WISC-IV^INDIA^ and the total CVS score. We also calculated a combined NEPSY-II z-score based on converted z-scores in the seven subtests. For the BRIEF 2, we used the overall Global Executive Composite score in the analyses. Children’s height-for-age z-score (HAZ) at follow up was calculated based on WHO growth standards [30]. The wealth of an individual was determined by a wealth index created using principal component analysis based on assets owned by the household [31]. Using the score from the principal component analysis, the population was divided into five equal wealth quintiles i.e. poorest, very poor, poor, less poor and least poor.

We used multivariable linear regression to investigate the association between vitamin D status at baseline and the cognitive outcomes and HAZ score at follow up. We used generalized linear model (GLM) with the gaussian distribution family, and identity link function to calculate beta-coefficients for the cognitive outcomes and the HAZ scores. We used GLM with the poisson distribution family, and log link to calculate the relative risk (RR) for stunting [32].

We used a method of purposeful selection of covariates to identify variables for the multivariable models [33, 34]. We included in the multivariable models the variables that changed the beta coefficient or relative risk of the outcome variables by 20% from the univariable models. We present the adjusted models, including variables that were identified in the process. The candidate variables for these models were age and sex of the child, maternal and paternal years of schooling, paternal occupation, wealth quintiles at follow up and baseline log (base e) cobalamin, folate, and total homocysteine concentration and the intervention groups.

Statistical analyses were performed using STATA version 15 (Stata Corporation, College Station, TX). We used generalized additive models in the statistical software R version 3.1.2 (The R Foundation for Statistical Computing, Vienna, Austria) to explore nonlinear associations between the plasma vitamin D concentration at baseline and the combined WISC-IV^INDIA^ and CVS z-score, the combined NEPSY-II z-score and the Global BRIEF-2 score at follow up after adjustment for potential confounders [35].

## Results

Of the 1000 children in the main study, 791 children consented to participate in the follow-up study. Fig. 1 shows the flow of the participants. The demographic information and nutritional status of the children at baseline and follow up are presented in Table 1. Baseline vitamin D status was available for 716 children who consented. Of these, 328 (45.8%) were deficient, 234 (32.7%) were inadequate, and 154 (21.5%) sufficient [29]. The mean (SD) and median (IQR) of vitamin D concentration at baseline were 14.6 (8.6) ng/ml and 12.8 (8.3–18.7) ng/ml, respectively.

**Table 1:** Demographic information and nutritional status of the 716 North Indian children at baseline (6 to 30 months) and follow up (6 to 9 years)

	Vitamin D sufficient (*n* = 154)	Vitamin D inadequate (*n* = 234)	Vitamin D deficient (*n* = 328)
Child characteristics at baseline (6 to 30 months)			
Boys, n (%)	85 (55.2)	125 (53.4)	161 (49.1)
Ever breastfed, n (%)	154 (100)	232 (99.1)	323 (98.5)
Growth Z scores, mean (sd)			
Weight-for-height (WHZ)	−0.9 (0.9)	− 0.9 (0.9)	− 0.8 (0.9)
Height-for-age (HAZ)	−1.7 (1.1)	− 1.6 (1.1)	− 1.6 (1.2)
Weight-for-age (WAZ)	− 1.6 (0.9)	−1.5 (1)	− 1.5 (1)
Wasted (<−2 WHZ)	18 (11.7)	28 (12)	34 (10.4)
Stunted (<−2 HAZ)	59 (38.3)	86 (36.7)	128 (39)
Underweight (<−2 WAZ)	47 (30.5)	77 (32.9)	105 (32)
Biomarkers:			
Cobalamin, mean (sd) pmol/L	312.7 (186.7)	332.6 (215.3)	305.9 (175.2)
Cobalamin < 200 pmol/L, n (%)	47 (30.5)	72 (30.8)	106 (32.3)
Folate, mean (sd) nmol/L	16.7 (14.6)	16.1 (13.7)	14.9 (14.4)
Folate < 7.5 nmol/L, n (%)	48 (31.2)	69 (29.5)	115 (35.1)
Homocysteine, mean (sd) μmol/L	14.4 (8.8)	13.3 (6.7)	13.7 (7.3)
Homocysteine > 10 μmol/L, n (%)	104 (68.4)	144 (62.1)	204 (62.4)
Soluble transferrin receptor, mean (sd) nmol/L	4.3 (2.8)	4.4 (3.3)	4.7 (3.1)
Soluble transferrin receptor> 4.7 nmol/L, n (%)	40 (26)	76 (32.5)	111 (33.8)
Child characteristics at follow up (6 to 9 years)			
Age follow up (yrs) mean (SD)	7.9 (0.6)	7.8 (0.6)	7.9 (0.6)
Schooling			
No School, n (%)	3 (1.9)	6 (2.6)	4 (1.2)
Hindi medium, n (%)	61 (39.6)	88 (37.6)	129 (39.3)
English medium, n (%)	90 (58.4)	140 (59.8)	195 (59.4)
Family characteristics at follow up			
Mothers years of schooling			
No schooling, n (%)	54 (35.5)	65 (28)	76 (23.3)
Primary (1–5 years), n (%)	23 (15.1)	18 (7.8)	49 (15)
Middle (6–12 years), n (%)	65 (42.8)	123 (53)	153 (46.9)
Higher (> 12 years), n (%)	10 (6.6)	26 (11.2)	48 (14.7)
Fathers occupation			
Government job or private services, n (%)	88 (57.1)	135 (57.9)	165 (50.8)
Self-employed, n (%)	31 (20.1)	56 (24)	95 (29.2)
Daily wager/farming, n (%)	29 (18.8)	31 (13.3)	52 (16)
No job/other, n (%)	6 (3.9)	11 (4.7)	13 (4)
Wealth Quintile			
Poorest, n (%)	39 (25.3)	47 (20.1)	60 (18.3)
Very Poor, n (%)	36 (23.4)	38 (16.2)	65 (19.8)
Poor, n (%)	31 (20.1)	57 (24.4)	60 (18.3)
Less Poor, n (%)	32 (20.8)	43 (18.4)	69 (21)
Least Poor, n (%)	16 (10.4)	49 (20.9)	74 (22.6)

The estimates from both univariable and multivariable analyses comparing the combined WISC-IV^INDIA^ and CVS z-score, the combined NEPSY-II z-score and the Global BRIEF-2 score between vitamin D inadequate, deficient, and vitamin D sufficient children are shown in Table 2. There were no significant differences between the vitamin D sufficient children, inadequate and deficient children on any of the cognitive outcomes.

**Table 2:** The association between baseline Vitamin D status and cognitive scores at follow up in North Indian children 6 to 9 years

	WISC-IV^INDIA^ and CVS z-score		NEPSY z-score		Global BRIEF score	
	Unadjusted β coefficient (95% CI)	Adjusted β coefficient (95% CI)^a^	Unadjusted β coefficient (95% CI)	Adjusted β coefficient (95% CI)^a^	Unadjusted β coefficient (95% CI)	Adjusted β coefficient (95% CI)^a^
Vitamin D sufficient	**Reference**		**Reference**		**Reference**	
Vitamin D inadequate	0.03 (−0.17 to 0.23)	−0.12 (−0.30 to 0.05)	−0.01 (−0.21 to 0.19)	−0.15 (−0.33 to 0.04)	0.21 (−2.10 to 2.48)	0.87 (−1.40 to 3.14)
Vitamin D deficient	0.02 (− 0.16 to 0.22)	− 0.13 (− 0.29 to 0.04)	−0.02 (− 0.21 to 0.17)	−0.16 (− 0.34 to 0.02)	−1.10 (−3.21 to 1.05)	−0.38 (− 2.53 to 1.77)

^a^adjusted for log folate, log soluble transferrin receptor and log homocysteine level at baseline, and the wealth index, paternal occupational status and maternal education at follow-up and intervention group

Table 3 shows the association between baseline vitamin D status and linear growth at follow up. Of the children, 15.8, 12.4 and 17.5% were stunted in the vitamin D deficient, inadequate and sufficient group, respectively. Vitamin D status was not associated with the HAZ score or the proportion of children stunted at follow up.

**Table 3:** The association between baseline Vitamin D status and linear growth at follow up in North Indian children 6 to 9 years

	HAZ scores at follow up		Stunted at follow up	
	Unadjusted β coefficient (95% CI)	Adjusted β coefficient (95% CI)*	Unadjusted RR (95% CI)	Adjusted RR (95% CI)^a^
Vitamin D sufficient	**Reference**		**Reference**	
Vitamin D inadequate	0.06 (−0.13 to 0.26)	−0.07 (− 0.26 to 0.12)	0.70 (0.42 to 1.20)	0.87 (0.50 to 1.50)
Vitamin D deficient	0.08 (−0.10 to 0.27)	−0.06 (− 0.24 to 0.11)	0.90 (0.57 to 1.44)	1.14 (0.70 to 1.86)

^a^adjusted for log folate, log soluble transferrin receptor and log homocysteine level, at baseline and the wealth index, paternal occupational status and maternal education at follow-up and intervention group

**Fig. 2:**
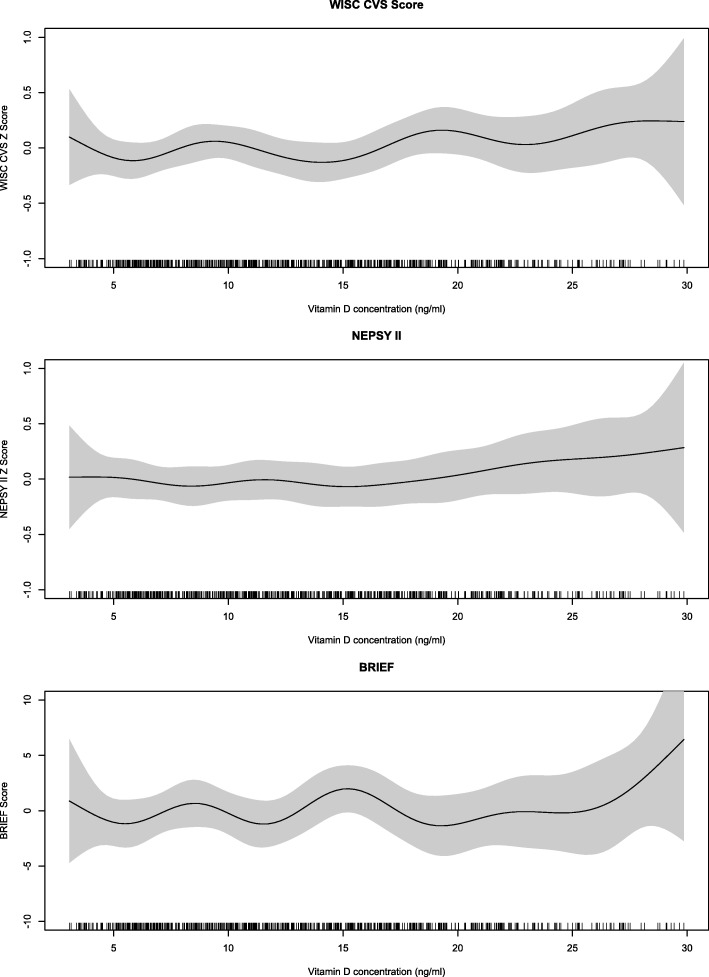
Association between baseline vitamin D level and the combined WISC IV^INDIA^ and Crichton Vocabulary Scale (CVS) z-score, the combined NEPSY II z scores and the BRIEF P Global Executive composite score at follow up in North Indian children 6 to 9 years. The graph was constructed using generalized additive models in R, the solid line depicts the association of vitamin D level at baseline and Global BRIEF score at follow up. The shaded area spans the 95% confidence interval of this association

The association between vitamin D concentration at baseline and the cognitive outcomes at follow up are depicted in Fig. 2. The GAMs did not reveal any non-linear associations between vitamin D level at baseline and the combined WISC-IV^INDIA^ and CVS z-score and the Global BRIEF-2 score at follow up.

## Discussion

We did not find any evidence for a link between early vitamin D status and long-term cognition and linear growth.

To our knowledge, this is the first study that has examined the relationship between vitamin D status in early childhood and cognitive development at school age. The findings from this study are in accordance with findings from the same cohort when neurodevelopment was measured in early childhood [13]. The findings are also similar to those from a cohort study in India, where vitamin D status in early childhood was not associated with the gross motor functioning among school aged children [36]. Studies that have examined the association between cord blood vitamin D concentrations and neurodevelopment measured in early and middle childhood have shown mixed results [37, 38]. Furthermore, studies that have examined the associations between vitamin D deficiency during pregnancy and neurodevelopment during early and middle childhood have also shown inconsistent results [7–12, 39]. Three studies found an association between pregnancy vitamin D status and neurodevelopment outcomes in children before 4 years of age [8, 11, 38], while one study found marginal associations with language scores at 10 years of age [10]. The inconsistencies are likely due to differences in the populations, the timing of vitamin D assessment during pregnancy, the use of different cut-offs for vitamin D deficiency, age of the child at developmental assessments, and the way potential confounders were handled. The large sample size, the broad range of cognitive assessments, many with Indian norms, and the timing of the assessments at school age provide strengths to our findings that early vitamin D status most likely is not associated with cognitive function on long term.

We found no association between vitamin D status at baseline and linear growth at follow up. There are similar findings described in pre-school children in Nepal and in uninfected HIV exposed infants in Africa [40, 41]. In contrast, low-birth-weight (1.8 to 2.5 kg) infants in India, who received 1 RDA of vitamin D supplementation showed significantly higher length and weight at 6 months of age compared to those who received placebo [42]. Vitamin D helps the growth plate cells to be more sensitive to growth hormone action which plays an important role in linear growth at school age [43]. Vitamin D also maintains bone health to ensure normal calcium and phosphate levels in the blood [44]. Thus, our findings of no association between vitamin D deficiency and linear growth may be unexpected. A probable explanation could be that the children have to be severely deficient in vitamin D before it has consequences for their bone growth. There may be deficiencies of other growth-limiting macro and micronutrients such as calcium, zinc, vitamin B12 that account for the variance of growth between these study children. Furthermore, lower proportion of animal source protein in food, may also contribute to poor growth in this population [45]. The role of vitamin D might thus be negligible in the light of other growth limiting factors in this population.

Dietary sources of vitamin D are limited mainly to oily fish, eggs, and fortified foods [46]. As most people in the northern part of India are vegetarians, the predominant dietary source of vitamin D among the study children would be milk. Milk is rarely fortified with vitamin D in India and the vitamin D content of unfortified milk is very low (2 IU/100 mL). Prevalence of lactose intolerance also contributes to vitamin D deficiency in this setting [47]. Currently, there is no national program of vitamin D supplementation for infants and children, but the Indian Academy of Pediatrics guidelines recommend daily vitamin D supplementation in doses of 400 IU up to 1 year of age and 600 IU from 1 to 18 years of age [48].

The main strength of the study is that we measured vitamin D status in a large sample of children during what is considered a critical window for the brain development, and measured the cognitive outcomes during a period where valid and stable estimates can be obtained. The study includes high quality and comprehensive assessment of cognitive development, with the use of validated tests with Indian norms. We were able to include 80% of the children from the primary cohort after more than 5 years with no significant differences between the children who were included in the follow-up and not. An immunoassay method was used to assess vitamin D concentration. The immunoassay can underestimate serum 25(OH) D2 concentration compared to liquid chromatography-tandem mass spectrometry (LC-MS/MS) [49].

## Conclusion

The results from the current study do not support that vitamin D status in early childhood is of importance for long-term growth and cognition.

## Data Availability

Request for data pertaining to the current analysis may be sent to Dr. Sunita Taneja (Email id: sunita.taneja@sas.org.in).

## Abbreviations

BRIEF: Behavior rating inventory of executive function
CI: Confidence interval
CVS: Crichton Verbal Scale
NEPSY: A developmental neuro psychological assessment
RCT: Randomized controlled trial
RDA: Recommended daily allowance
SD: Standard deviation
tHcy: Total Homocysteine
WISC-IV ^INDIA^: Wechsler intelligence scale for children, 4th edition ^INDIA^
